# A Stochastic Markov Chain Model to Describe Lung Cancer Growth and Metastasis

**DOI:** 10.1371/journal.pone.0034637

**Published:** 2012-04-27

**Authors:** Paul K. Newton, Jeremy Mason, Kelly Bethel, Lyudmila A. Bazhenova, Jorge Nieva, Peter Kuhn

**Affiliations:** 1 Department of Aerospace & Mechanical Engineering and Department of Mathematics, University of Southern California, Los Angeles, California, United States of America; 2 Scripps Clinic Torrey Pines, La Jolla, California, United States of America; 3 UCSD Moores Cancer Center, La Jolla, California, United States of America; 4 Billings Clinic, Billings, Montana, United States of America; 5 The Scripps Research Institute, La Jolla, California, United States of America; University of Pittsburgh, United States of America

## Abstract

A stochastic Markov chain model for metastatic progression is developed for primary lung cancer based on a network construction of metastatic sites with dynamics modeled as an ensemble of random walkers on the network. We calculate a transition matrix, with entries (transition probabilities) interpreted as random variables, and use it to construct a circular bi-directional network of primary and metastatic locations based on postmortem tissue analysis of 3827 autopsies on untreated patients documenting all primary tumor locations and metastatic sites from this population. The resulting 50 potential metastatic sites are connected by directed edges with distributed weightings, where the site connections and weightings are obtained by calculating the entries of an ensemble of transition matrices so that the steady-state distribution obtained from the long-time limit of the Markov chain dynamical system corresponds to the ensemble metastatic distribution obtained from the autopsy data set. We condition our search for a transition matrix on an initial distribution of metastatic tumors obtained from the data set. Through an iterative numerical search procedure, we adjust the entries of a sequence of approximations until a transition matrix with the correct steady-state is found (up to a numerical threshold). Since this constrained linear optimization problem is underdetermined, we characterize the statistical variance of the ensemble of transition matrices calculated using the means and variances of their singular value distributions as a diagnostic tool. We interpret the ensemble averaged transition probabilities as (approximately) normally distributed random variables. The model allows us to simulate and quantify disease progression pathways and timescales of progression from the lung position to other sites and we highlight several key findings based on the model.

## Introduction

The identification of circulating tumor cells (CTCs) in the human circulatory system dates back to Ashworth’s 1869 paper [1] in which he identified and pointed out the potential significance of cells similar to those found in the primary tumor of a deceased cancer victim. Since then, there has been sporadic focus on CTCs as a key diagnostic tool in the fight against cancer, based mostly on the so-called ‘seed and soil’ hypothesis [2]–[4] of cancer metastasis, in which the CTCs play the role of seeds which detach from the primary tumor, disperse through the bloodstream, and get trapped at various distant sites (typically small blood vessels of organ tissues), then, if conditions are favorable, extravasate, form metastases, and subsequently colonize. The metastatic sites offer the soil for potential subsequent growth of secondary tumors. Paget’s 1889 seed-and-soil hypothesis [3] asserts that the development of secondary tumors is not due to chance alone, but depends on detailed interactions, or cross-talk, between select cancer cells and specific organ microenvironments. In 1929, J. Ewing challenged the seed-and-soil hypothesis [5] by proposing that metastatic dissemination occurs based on purely mechanical factors resulting from the anatomical structure of the vascular system, a proposal that is now known to be too simplistic an explanation for the metastatic patterns that are produced over large populations. While the seed-and-soil hypothesis remains a bedrock theory in cancer research, it has been significantly refined over the years to incorporate our current level of understanding on how the ability for a tumor cell to mestastasize depends on its complex interactions with the homeostatic factors that promote tumor cell growth, cell survival, angiogenisis, invasion, and metastastasis [2].

**Figure 1:**
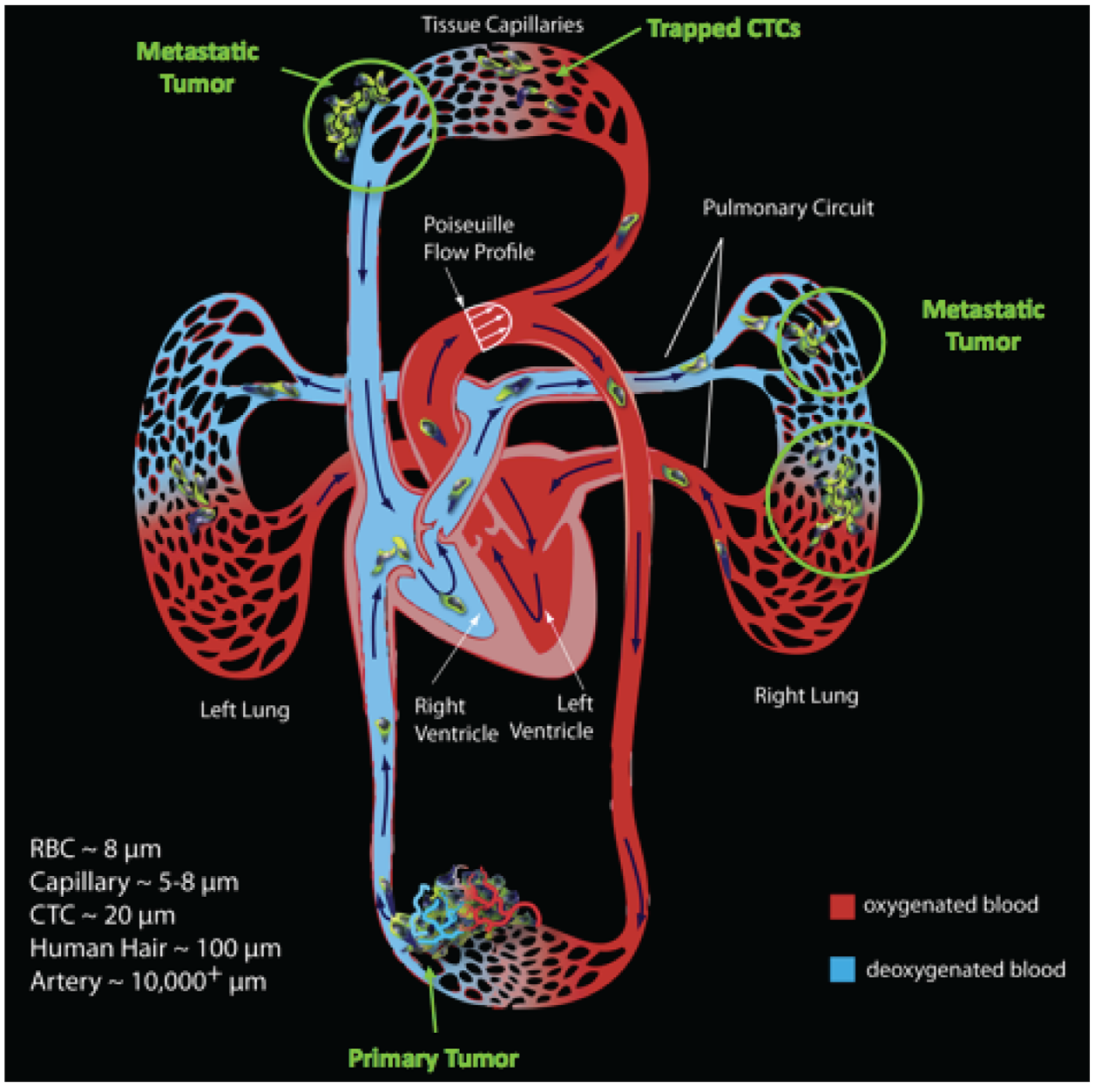
Schematic diagram of human circulatory system showing circulating tumor cells (CTCs) detaching from primary tumor and getting trapped in capillary beds and other potential future metastatic locations as outlined by the ‘seed-and-soil’ framework.

A schematic diagram associated with the metastatic process is shown in Figure 1. Here, the primary tumor (from which the CTCs detach) is located in the lower part of the diagram and the distant potential secondary locations where CTCs get trapped and form metastases are shown. In this paper, we will not be concerned with extravasation, colonization and the formation of secondary tumors which are complex processes in their own right [4], but rather with a probabilistic description of metastatic progression from primary neoplasm to metastatic sites; hence, we provide a quantitative framework for charting the time-evolution of cancer progression along with a stochastic description of the complex interactions of these cells with the organ microenvironment. Also shown in the figure are representative scales of a typical red blood cell (8 µm), capillary diameter (5–8 µm), CTC (20 µm), and human hair diameter (100 µm). The total number of remote sites at which metastases are found for any given type of primary cancer is relatively small (see the autopsy data set described in [6]), say on the order of 50 locations, those sites presumably being the locations at which CTCs get trapped and subsequently colonize. For any individual making up the ensemble, of course, the number of sites with metastatic tumors would be much smaller. A ‘ballpark’ estimate, based on the ratio of mets to primaries (from [6]) suggests a number around 9484/3827∼2.5, although in the modern era, this number is probably higher. A reasonably thorough overview of this process is described in [7].

It wasn’t until recently, however, that important technological developments in the ability to identify, isolate, extract, and genetically and mechanically study CTCs from cancer patients became available (see, for example [8]–[15]). These new approaches, in turn, produced the need to develop quantitative models which can predict/track CTC dispersal and transport in the circulatory and lymphatic systems of cancer patients for potential diagnostic purposes. As a rough estimate, data (based primarily on animal studies) shows that within 24 hours after release from the primary tumor, less than 0.1% of CTCs are still viable, and fewer than those, perhaps only a few from the primary tumor, can give rise to a metastasis. There are, however, potentially hundreds of thousands, millions, or billions of these cells detaching from the primary tumor continually over time [16], [17], and we currently do not know how to deterministically predict which of these cells are the future seeds, or where they will take root. All of these estimates, along with our current lack of detailed understanding of the full spectrum of the biological heterogeneity of cancer cells, point to the utility of a statistical or probabilistic framework for charting the progression of cancer metastasis. This is a particularly important step for any potential future comprehensive computer simulation of cancer progression, something not currently feasible. Although the dispersion of CTCs is the underlying dynamical mechanism by which the disease spreads, the probabilistic framework obviates the need to model all of the biomechanical features of the complex processes by which cells journey through the vascular/lymphatic system. This paper provides the mathematical/computational framework for such an approach.

In this paper, we develop a new Markov chain based model of metastatic progression for primary lung cancer, which offers a probabilistic description of the time-history of the disease as it unfolds through the metastatic cascade [4]. The Markov chain is a dynamical system whose state-vector is made up of all potential metastatic locations identified in the data set described in [6] (defined in Table 1), with normalized entries that can be interpreted as the time-evolving (measured in discrete steps *k*) probability of a metastasis developing at each of the sites in the network. One of the strengths of such a statistical approach is that we need not offer specific biomechanical, genetic, or biochemical reasons for the spread from one site to another, those reasons presumably will become available through more research on the interactions between CTCs and their microenvironment. We account for all such mechanisms by defining a transition probability ( which is itself a random variable) of a random walker dispersing from one site to another, thus creating a quantitative and computational framework for the seed-and-soil hypothesis as an ensemble based first step, then can be further refined primarily by using larger, better, and more targeted databases such as ones that focus on specific genotypes or phenotypes, or by more refined modeling of the correlations between the trapping of a CTC at a specific site, and the probability of secondary tumor growth at that location.

**Table 1:** Metastatic site numbering system.

#	Name	#	Name
1	Adrenal*	26	Omentum*
2	Anus	27	Ovaries
3	Appendix	28	Pancreas*
4	Bile Duct	29	Penis
5	Bladder	30	Pericardium*
6	Bone*	31	Peritoneum*
7	Brain*	32	Pharynx
8	Branchial Cyst	33	Pleura*
9	Breast	34	Prostate*
10	Cervix	35	Rectum
11	Colon	36	Retroperitoneum
12	Diaphragm*	37	Salivary
13	Duodenum	38	Skeletal Muscle*
14	Esophagus	39	Skin*
15	Eye	40	Small Intestine*
16	Gallbladder*	41	Spleen*
17	Heart*	42	Stomach*
18	Kidney*	43	Testes
19	Large Intestine*	44	Thyroid*
20	Larynx	45	Tongue
21	Lip*	46	Tonsil
22	Liver*	47	Unknown
23	Lung*	48	Uterus*
24	Lymph Nodes (reg)*	49	Vagina*
25	Lymph Nodes (dist)*	50	Vulva

Site numbering system used in transition matrix and network model. The ^*^ indicates an entry in the target vector associated with lung cancer primary from the data set of [6].

**Figure 2:**
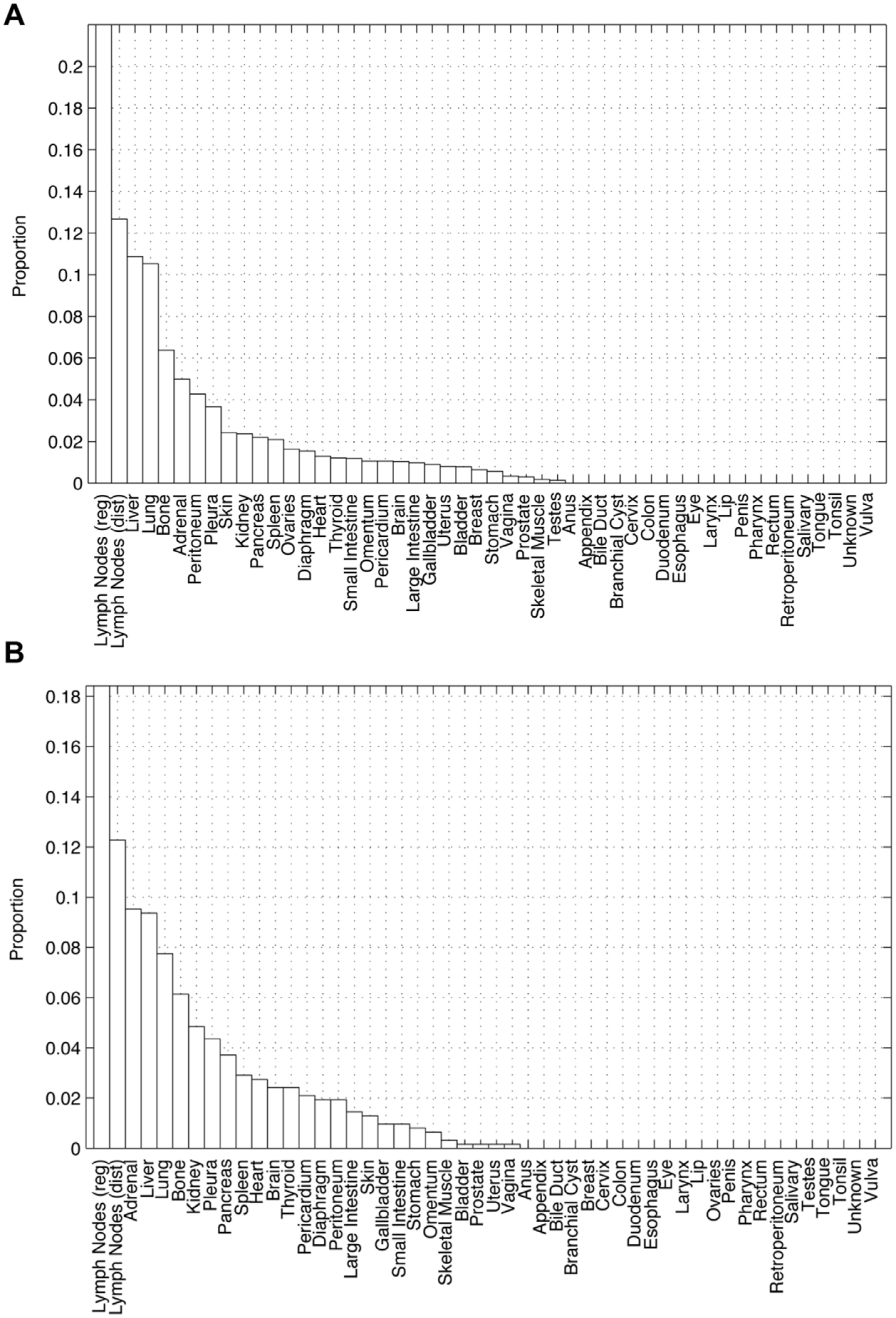
Metastatic distributions from autopsy data set extracted from 3827 patients [6]. Y-axis in each graph represents a proportion between 0 and 1. The sum of all the heights is 1. These are the two key probability distributions used to ‘train’ our lung cancer progression model. (a) Overall metastatic distribution including all primaries. We call this distribution the ‘generic’ distribution as it includes all primary cancer types.; (b) Distribution of metastases associated with primary lung cancer. We call this distribution the ‘target’ distribution that we label 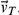

The Markov chain dynamical system takes place on a metastatic network based model of the disease, which we calculate based on the available data over large populations of patients. In particular, we use the data described in the autopsy analysis in [6] in which metastatic distributions in a population of 3827 deceased cancer victims were analyzed. None of the victims received chemotherapy or radiation. The autopsies were performed between 1914 and 1943 at 5 separate affiliated centers, with an ensemble distribution of 41 primary tumor types, and 30 metastatic locations. Figure 2 shows histograms of the number of metastases found at the various sites in the population. Figure 2(a) shows the metastatic distribution in the entire population, while Figure 2(b) shows the distribution in the subset of the population with primary lung cancer. We note that this data offers no particular information on the time history of the disease for the population or for individual patients - only the long-time metastatic distribution in a population of patients, where long-time is associated with end of life, a timescale that varies significantly from patient to patient (even those with nominally the same disease). Although this paper focuses on a model for primary lung cancer, the approach would work equally well for all of the main tumor types. Indeed, one of the goals of future studies will be to compare the models obtained for different cancer types.

Network based models of disease progression have been developed recently in various contexts such as the spread of computer viruses [18], general human diseases [19], and even cancer metastasis [20], but as far as we are aware, our Markov chain/random walk approach to modeling the dynamics of the disease on networks constructed for each primary cancer type from patient populations offers a new and potentially promising computational framework for simulating disease progression. More general developments on the structure and dynamics on networks can be found in the recent works [21]–[26]. For brief introductions to some of the mathematical ideas developed in this paper, see [27]–[30].

## Results

In this section we describe three main results from the model. First, the model separates the 27 non-zero sites from Figure 2(b) into what we call ‘first-order’ sites (20 of these), and ‘second-order’ sites (7 of these). Second, the model quantifies the ability of each site to self-seed by ranking the average edge weight of each site back to itself (see [31]). Of these, the strongest self-seeders are the lymph nodes, bone, kidney, and lung. Third, the model allows us to calculate a time-ordering (model based) associated with metastatic progression. This is achieved by performing Monte Carlo simulations of the mean first-passage times from the lung site to each of the other sites in the network. The mean first-passage time is the average number of edges a random walker must traverse in order to hit a given site, hence the number is not restricted to take on discrete integer values. We think of these mean first-passage times as the proxy timescale for progression. In principle, they can be calculated analytically using the fundamental matrix (see [32]), but in practice, since this involves inverting the 50×50 transition matrix, it is far more convenient to obtain the results numerically via Monte Carlo simulations. The results will be described in terms of a ‘random walker’ leaving the lung site and traversing the network, moving from site to site along one of the outgoing edges available to it at the site it is leaving, choosing a given edge with the probability corresponding to its weighting.

### Description of the Markov Chain Model

With the stochastic transition matrix 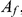 we briefly describe the basic features and interpretations of a Markov dynamical system model which we write as:
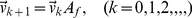
(1)


The matrix 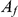 is our transition matrix which is applied to a state-vector 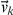 at each discrete time-step *k* to advance to step 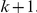 Thus, it is easy to see that:
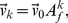
(2)where 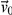 is the initial-state vector. The underlying dynamics associated with disease progression is interpreted as a random walk on the weighted directed network defined by the entries of the transition matrix.

### The State Vectors and Definition of the Steady-state

To interpret the meaning of the initial-state vector and the transition matrix, one should think of the patient’s initial tumor distribution in terms of probabilities, or ‘uncertanties’. Thus, an initial-state vector with a 1 in the 23rd entry:
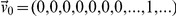
in our 50 node model indicates, with absolute certainty, that the patient has a primary tumor located in the ‘lung’ (position 23). At the other extreme, we may have an initial-state vector:



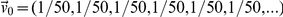
which indicates that all locations of the initial tumor distribution are equally likely. One interpretation of this is that we have no information at all about where the primary tumor is located. A third possibility is that we have *some* limited information about the initial tumor distribution, but not completely certain information, thus an initial-state vector:



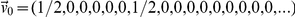
would indicate that we think it likely that there is a primary tumor in the ‘adrenal’ (position 1), or ‘brain’ (position 7), but we are not sure which.

Then, we can ask how this initial information propagates forward in time as the disease progresses. To advance one-step forward in time, we apply the transition matrix once to the initial-state vector, thus:
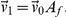



This gives us our new state-vector 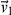 after step one. For the next step, we apply the transition matrix again, this time to 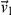:
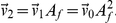



The dynamical system proceeds according to eqns (2) in a manner consistent with the schematic diagram from Figure 1. As described in the introduction, it is best to think of the entries of the state-vector as probabilities for metastases developing at each of the discrete sites in our model (and in the data set), thus for the seed to take root in the soil. The entries of the state-vector 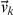 continually get redistributed in time, as measured in discrete steps *k*, until they reach the target steady-state distribution. A different interpretation of the entries of the state-vector at each discrete step is that they reflect the *ensemble statistical distribution* of a collection of agents executing a random walk across the network. We should point out, however, that for the ensemble of random-walkers all leaving from the lung site, the best way to measure the passage of time is via *mean first-passage times* to each of the sites, which we compute using Monte Carlo simulations. It is important to keep in mind that since the transition matrix is constructed based on an *autopsy* data set, there is no direct information available on time-histories of progression, only tumor distribution at time of death. A big advantage of using this data set is that we are able to build a model based on the ‘natural’ progression of the disease (i.e. untreated patients), whereas clinical data on time-histories of progression for untreated patients do not exist, as far as we are aware. Therefore, our challenge is to extract as much information as we can using the autopsy data set [6], keeping in mind that time should be interpreted only as the model timescale of progression. A next step would be to calibrate these model timescales with separate data sets containing time progression information, not something we consider in this paper.

Now comes a natural and important question. After long-times (*k* large), is there some steady-state distribution that is achieved by the model? Correspondingly, given a particular primary tumor, what are long-term probabilistic distributions of possible metastases? We call this distribution vector 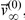 and define it as:
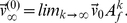
(3)


Notice that if a steady-state distribution is achieved, then for sufficiently large *k*, 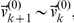 and since
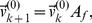
(4)this implies that



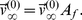
(5)Thus
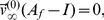
(6)which means that 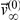 is a left-eigenvector of 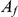 corresponding to eigenvalue 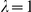. This is a crucial and practical observation that allows us to calculate the steady-state distribution 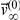 directly from the transition matrix. Since the rows of 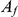 add to one, it always has at least one eigenvalue that is 1, hence there is always at least one steady-state distribution, but there may be more than one – this depends in detail on the matrix structure, something the eigenvalue distribution [40] can reveal.

**Figure 3:**
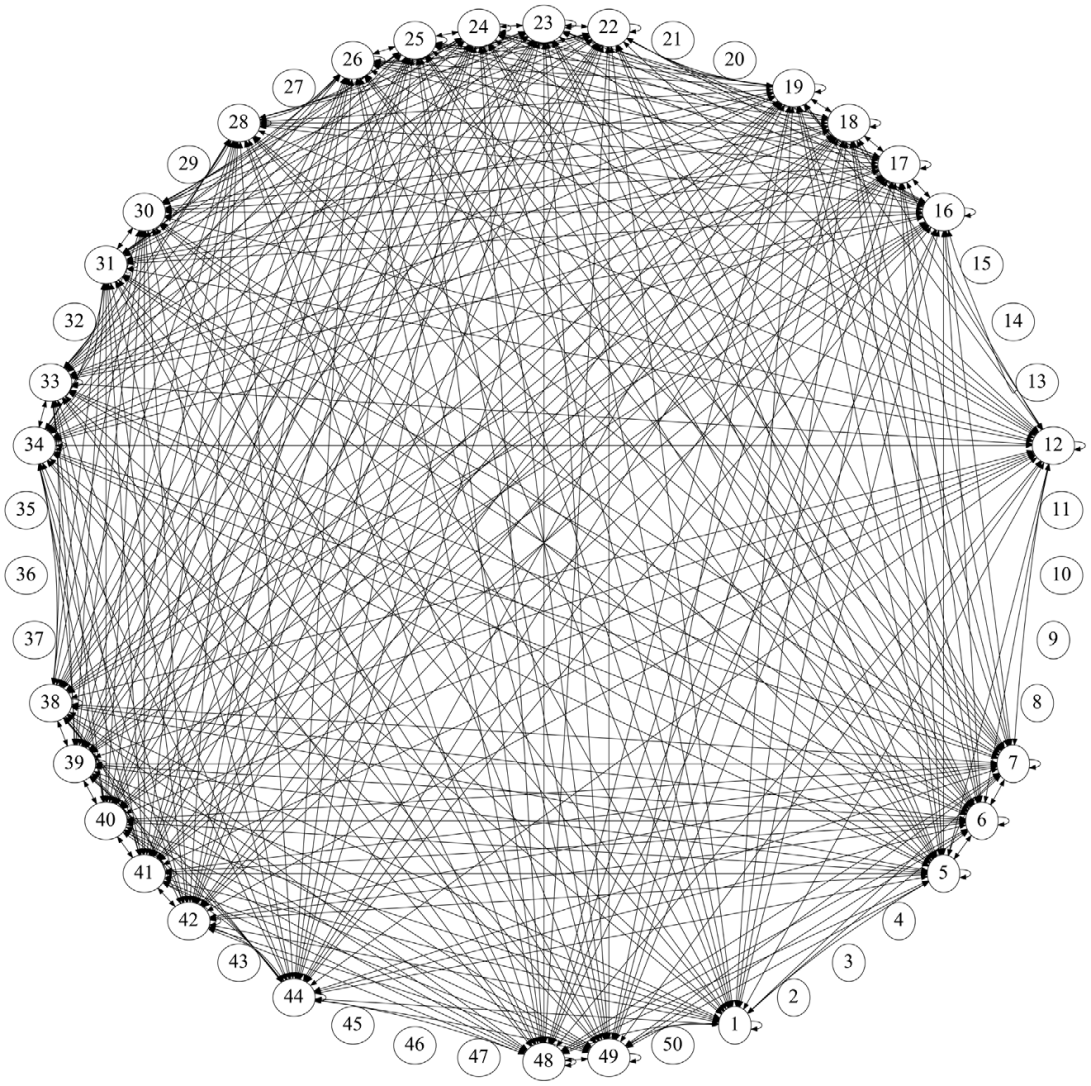
The converged lung cancer network shown as a circular, bi-directional, weighted graph. We use sample mean values for all edges connecting sites in the target distribution. The disease progresses from site 23 (lung) as a ‘random walker’ on this network. Arrow heads placed on the end or ends of the edges denote the direction of the connections. Edge weightings are not shown. There are 50 sites (defined in Table 1) obtained from the full data set of [6], with ‘Lung’ corresponding to site 23 placed on top. The 27 sites that are connected by edges are those from the target vector for lung cancer defined in Table 1.

The target distribution for lung cancer shown in Figure 2(b) and labeled 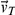 is not a steady-state for the matrix 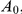 i.e.
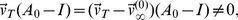
(7)since 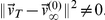


**Figure 4:**
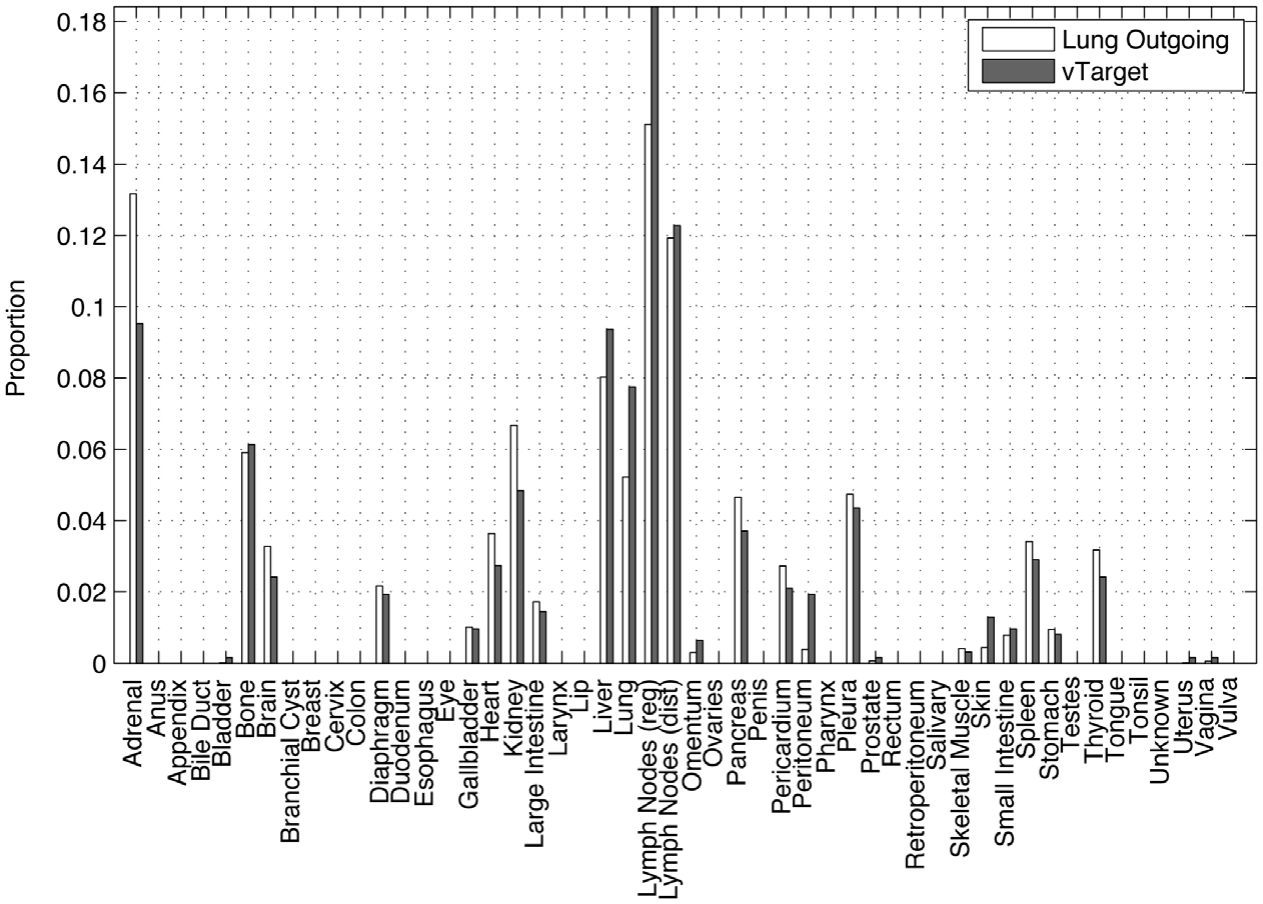
Weight of outgoing edges from the lung (using sample mean values from ensemble) as compared with the ‘target’ distribution.

**Figure 5:**
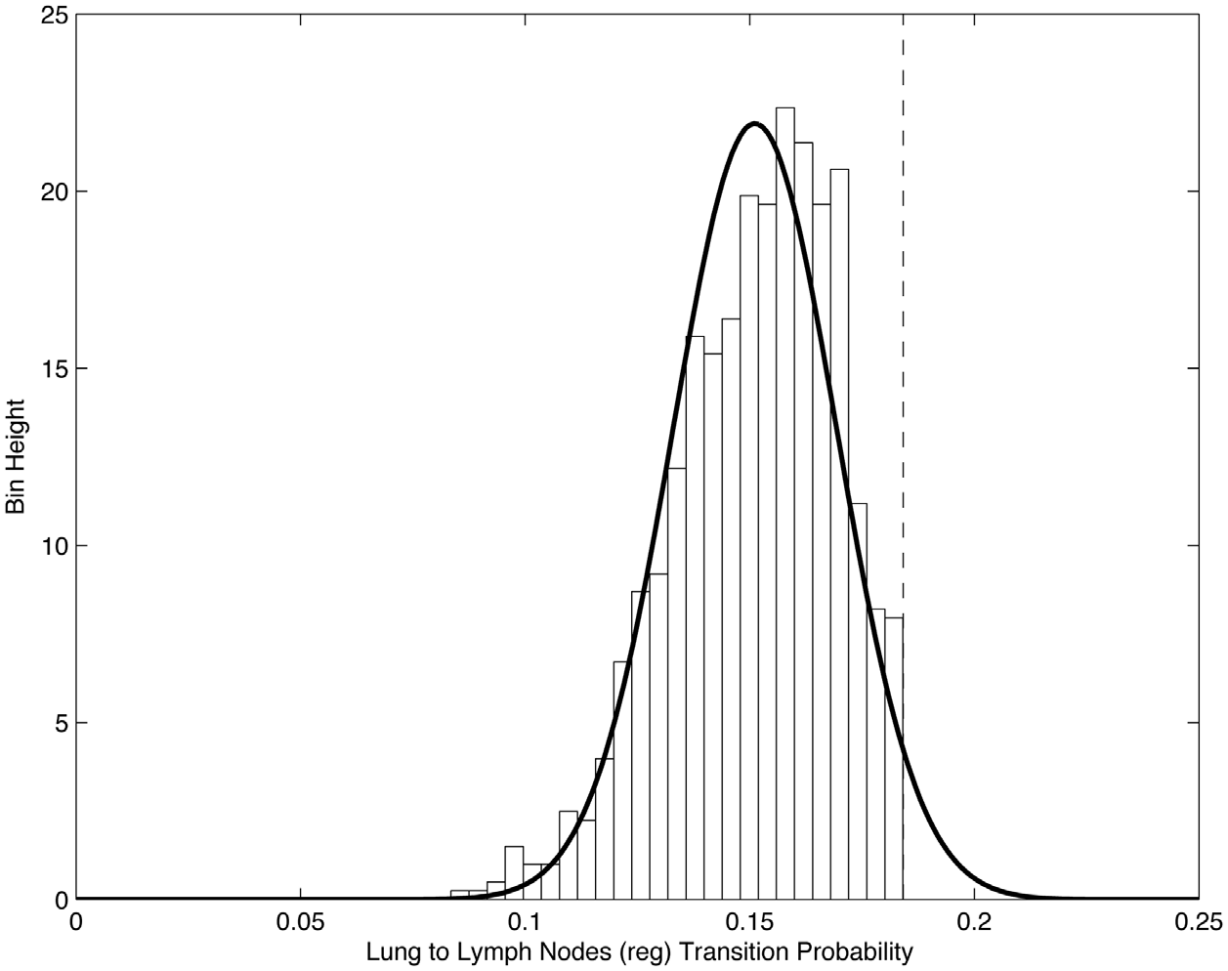
Histogram of edge values from lung to lymph nodes (reg) for 1000 trained 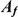
’s, showing that edge values (transition probabilities) are best thought of as random variables which are (approximately) normally distributed. Dashed vertical line shows initial edge value associated with 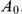 Normal distribution with sample mean (0.15115) and variance (0.01821) is shown as overlay.

### Structure of the Lung Cancer Matrix and Convergence to the Steady-state

Figure 3 shows the network diagram associated with the ensemble averaged converged matrix - this is the lung cancer network conditioned on our initial guess 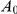 averaged over 1000 training sessions. Each of the sites has incoming and outgoing edges (denoted with arrow heads) which connect it to other sites in the target distribution where the cancer can spread, and each of the edges have a probabilistic weighting (not shown), with the constraint that the weightings associated with all outgoing edges at each site must sum to 1. The disease spreads across the network from an initial site following a random walk. To minimize the number of edges depicted in the figure, we have combined incoming and outgoing edges whenever possible, and placed arrow heads on both ends of an edge, instead of plotting the two edges separately.

**Figure 6:**
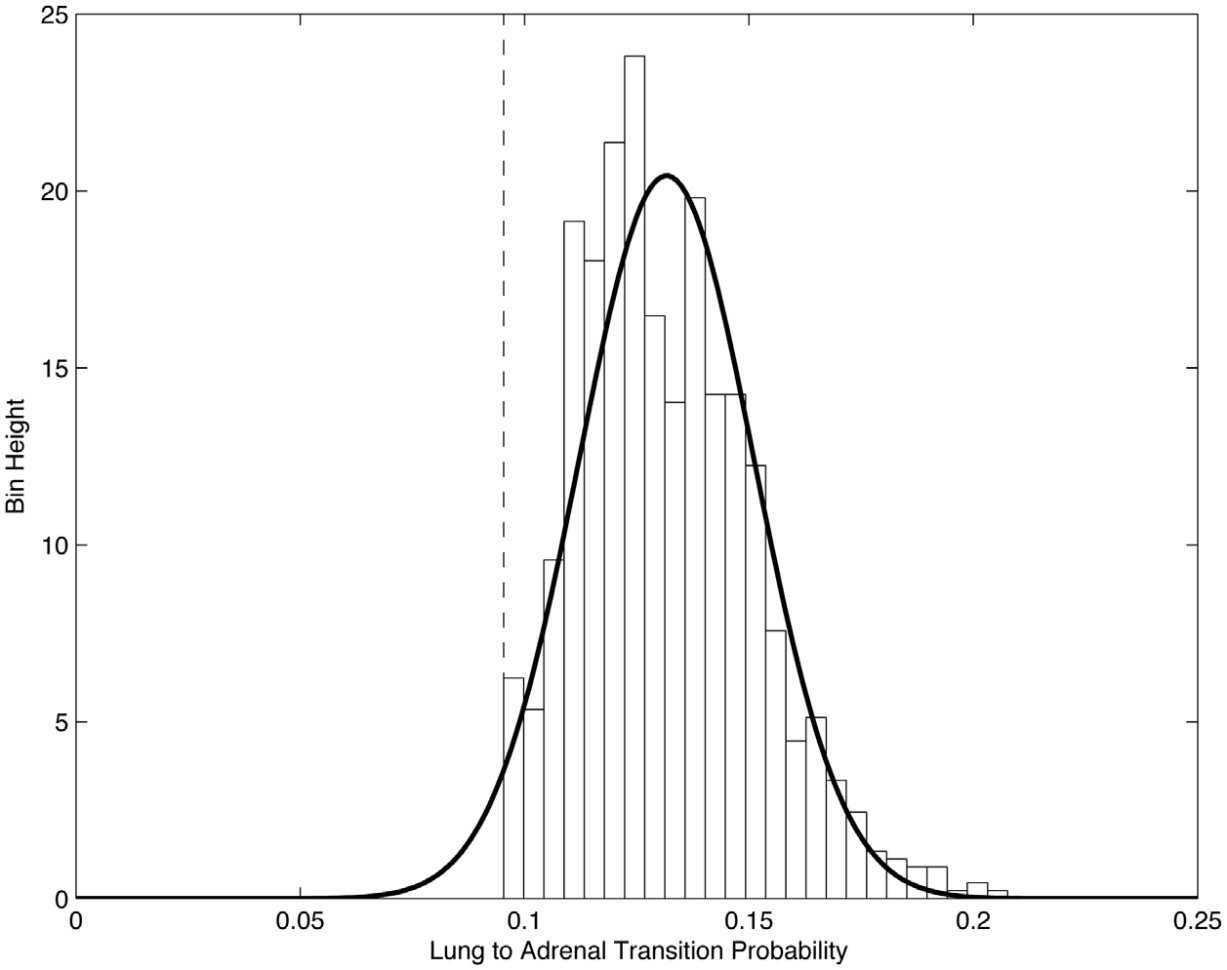
Histogram of edge values from lung to adrenal for 1000 trained 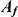
’s showing that edge values (transition probabilities) are best thought of as random variables which are (approximately) normally distributed. Dashed vertical line shows initial edge value associated with 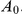 Normal distribution with sample mean (0.13165) and variance (0.01953) is shown as overlay.

In Figure 4 we plot the (mean) edge weightings of the outgoing edges from the lung, as compared with the values of the target distribution shown in Figure 2(b). The differences show that the values in the lung row of 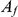 have adjusted from their initial values in 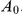 Figure 5 and Figure 6 highlight our interpretation of the transition probabilities, or edge values of the network, as random variables. We show in these figures the distributions associated with the ensemble of lung to regional lymph node (Figure 5) edge values, and those associated with the lung to adrenal (Figure 6) edge values. In each case, we histogram the edge values from the 1000 converged matrices, and use the sample means and variances to overlay a corresponding normal distribution. The vertical dashed lines in Figures 5 and 6 show the initial value of the transition probability from lung to regional lymph nodes (Figure 5) and lung to adrenal (Figure 6). These initial values used in the matrix 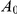 are obtained using the entire data set of DiSibio and French [6], i.e. over all primary cancer types. The converged Gaussian distributions shown in these figures, however, are specific to lung cancer only. The fact that the mean is clearly shifted to the left of the vertical line in Figure 5 indicates that the lung to regional lymph node connection for lung cancer is less significant, statistically, than for other cancer types. A possible anatomical explanation for this left shift could be the fact that regional lymph nodes, for lung cancer, are located very close to the lung itself, compared with their typical distance away from other primary tumor locations. Because of this unusually close proximity, regional lymph nodes could easily have been mistakingly considered as part of the lung in some of the autopsies in the series, effectively reducing the significance of the lung to regional lymph node connection. By contrast, the right shift of the mean, shown in Figure 6 for the lung to adrenal connection, would indicate that the lung to adrenal connection is statistically more important for lung cancer than for other primary cancer types. This could be due to the documented anatomic connection between lung and adrenal that is known, but has not, to date, been a particular focus of lung cancer metastasis studies.

**Figure 7:**
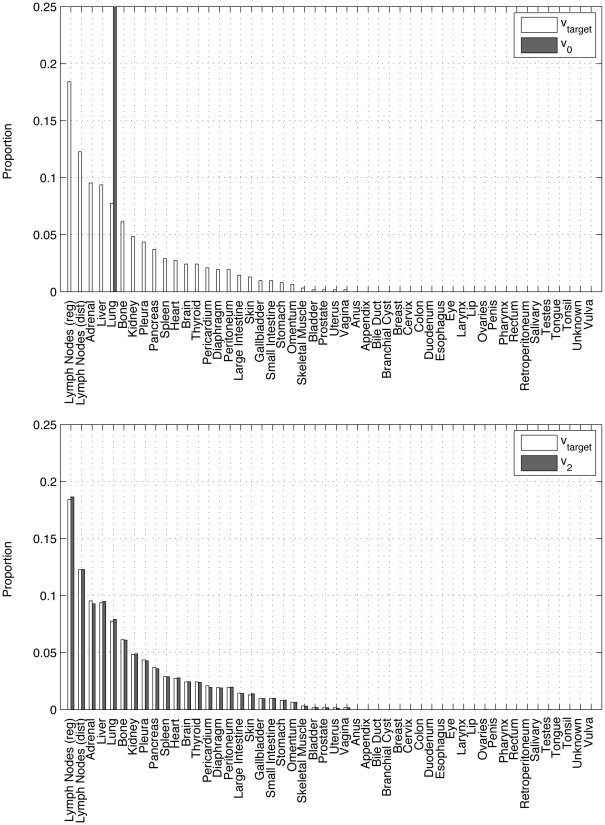
Panel showing progression of state vector 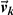 for lung cancer primary using the ensemble averaged lung cancer matrix. Filled rectangles show the long-time metastatic distribution from the autopsy data in Figure 2(b), unfilled rectangles show the distribution at step *k* using the Markov chain model. (a) *k* = 0; (b) *k* = 2.

**Figure 8:**
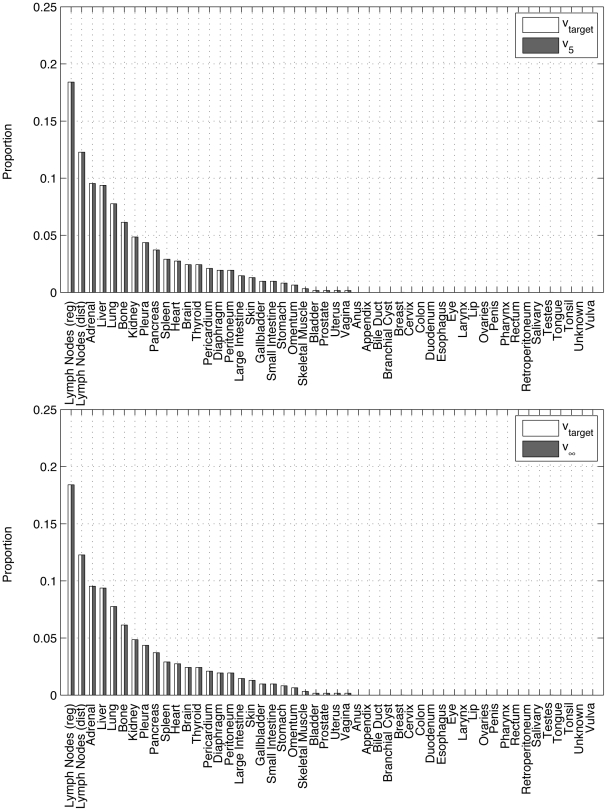
Panel showing progression of state vector 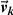 for lung cancer primary using the ensemble averaged lung cancer matrix. Filled rectangles show the long-time metastatic distribution from the autopsy data in Figure 2(b), unfilled rectangles show the distribution at step *k* using the Markov chain model. (a) *k* = 5; (b) *k* = ∞.

The dynamical system defined by the Markov process:
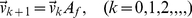
(8)can be thought of as governing the statistical distribution associated with random walkers traversing the network. Figures 7 and 8 show the dynamical progression of the initial state vector, starting with an initial state-vector corresponding to a lung tumor, i.e. 1 in position 23, with 0’s elsewhere. In the sequence, the target vector 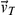 is depicted with filled bars, while the vector 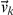 (for 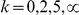) is depicted with unfilled bars. Convergence to the target is exponential. By *k* = 5, convergence to the steady-state is essentially complete.

**Table 2:** One and two-step transition probabilities.

Target Sites	One-step transitionprob (Avg)	Two-step transitionprobs
Lymph Nodes (reg)	0.15115±0.01821	0.02819 (LN (reg))
Adrenal	0.13165±0.01953	0.01397 (LN (reg))
Lymph Nodes (dist)	0.11928±0.00279	0.01860 (LN (reg))
Liver	0.08028±0.00946	0.01440 (LN (reg))
Kidney	0.06677±0.01231	0.00709 (LN (reg))
Bone	0.05914±0.00196	0.00931 (LN (reg))
Lung	0.05223±0.01504	0.01214 (LN (reg))
Pleura	0.04735±0.00338	0.00657 (LN (reg))
Pancreas	0.04660±0.00785	0.00549 (LN (reg))
Heart	0.03639±0.00739	0.00407 (LN (reg))
Spleen	0.03415±0.00454	0.00432 (LN (reg))
Brain	0.03274±0.00728	0.00360 (LN (reg))
Thyroid	0.03180±0.00628	0.00356 (LN (reg))
Pericardium	0.02733±0.00557	0.00306 (LN (reg))
Diaphragm	0.02169±0.00216	0.00289 (LN (reg))
Large Intestine	0.01724±0.00266	0.00219 (LN (reg))
Gallbladder	0.01015±0.00048	0.00145 (LN (reg))
Stomach	0.00949±0.00139	0.00119 (LN (reg))
Small Intestine	0.00786±0.00158	0.00149 (LN (reg))
Skeletal Muscle	0.00413±0.00093	0.00047 (LN (reg))
Skin	0.00439±0.00443	0.00203 (LN (reg))
Peritoneum	0.00384±0.00567	0.00308 (LN (reg))
Omentum	0.00305±0.00223	0.00103 (LN (reg))
Prostate	0.00064±0.00060	0.00025 (LN (reg))
Vagina	0.00052±0.00059	0.00025 (LN (reg))
Bladder	0.00009±0.00029	0.00023 (Adrenal)
Uterus	0.00007±0.00025	0.00022 (Adrenal)

The 27 target sites listed in decreasing order of their edge weights (ensemble average values) from lung site. The 20 sites above the ‘cut-off’ are called ‘First-Order’ sites. Their direct connections from the lung are strong enough so that they represent the most likely route to that site. The 7 sites listed below are called ‘Second-Order’ sites. Their connections from the lung are sufficiently weak that it is equally or more likely (taking into account standard deviations) to get to the site via some other first-order site (shown in parentheses).

### First and Second Order Sites

The 27 metastatic sites associated with lung cancer shown in the distribution of Figure 2(b) can be separated into two distinct groups in light of the ensemble averaged transition probabilities listed in decreasing order in Table 2. The middle column of this table shows the transition probability going directly from the lung to each of the 27 sites of the target vector (ensemble averaged±standard deviations). The right column of the table shows the most likely two-step path from lung to each of the sites listed on the left, via the most probable *intermediate* site. Thus it shows the product of the direct transition probability from lung to an intermediate site (in parentheses on right), times the transition probability from that intermediate site to the site listed on the left. When one compares these values (all are ensemble averaged) it is clear that the top 20 sites (listed above the cut-off line) have direct transition values higher than their most probable two-step transition, hence we call these ‘first-order’ sites. If the disease reaches one of these sites, the most likely path is directly from the lung after one-step. A random walker, leaving the lung site, after it chooses one of the available outgoing edges with probability corresponding to the edge weighting, will first visit one of these first-order sites. The most heavily weighted edges, hence the most likely first site visits, will be lymph nodes (reg) and adrenal, accounting for roughly 28% of the first-site visits. The next two most heavily weighted sites are lymph nodes (dist) and liver. These four sites account for roughly 50% of the first site visits of an ensemble of random walkers.

**Figure 9:**
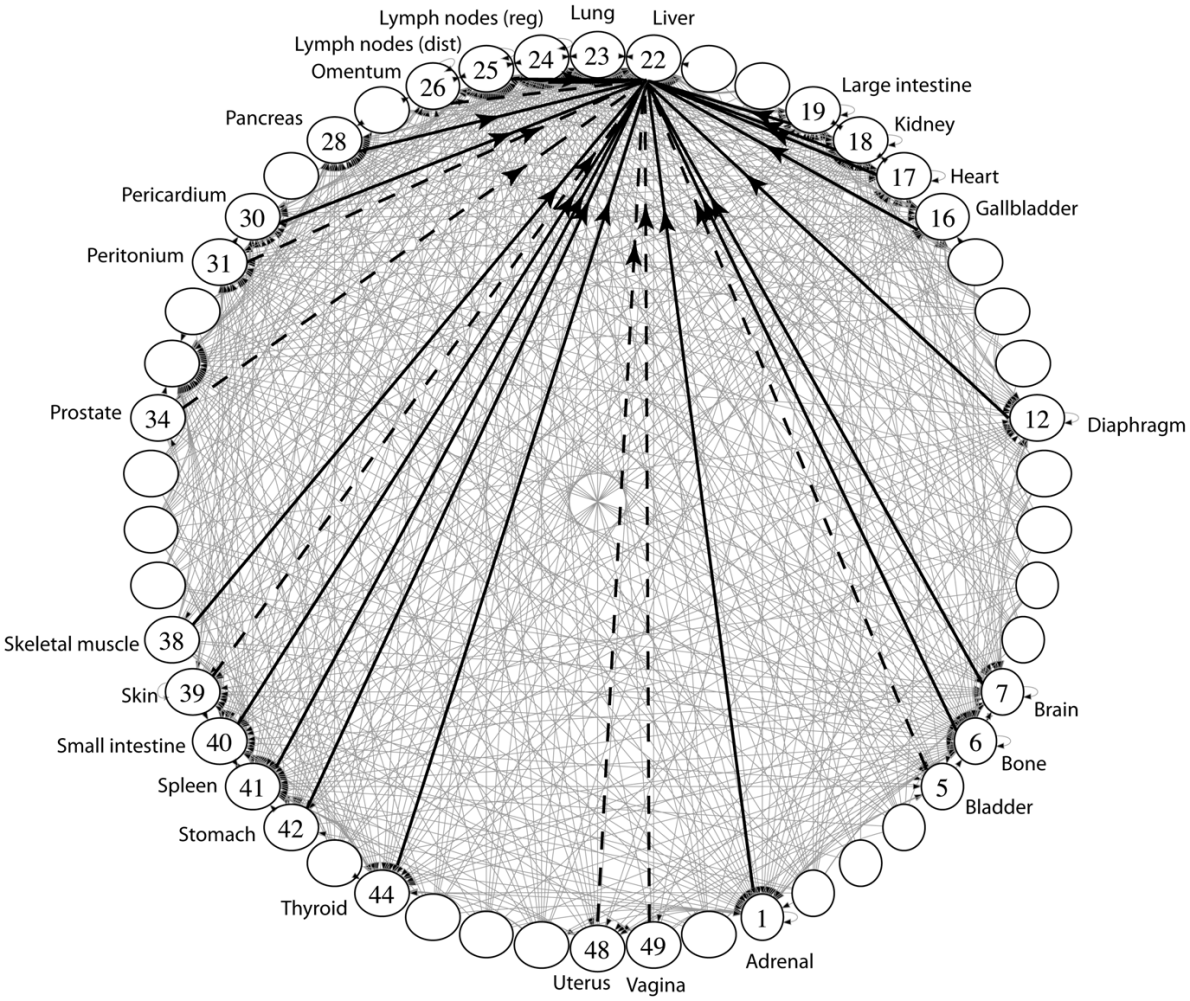
Probabilistic decomposition of pathways from lung to liver. First transition probability is directly from lung to liver (0.08028±0.00946). Paths from the first-order sites to liver are shown as solid arrows. Paths from second-order sites to liver are shown as dashed arrows.

**Table 3:** Self-edge weightings for each site.

Target Sites	Self-edge weight (avg)
Lymph Nodes (reg)	0.1865±0.0152
Lymph Nodes (dist)	0.1231±0.0028
Liver	0.0945±0.0094
Adrenal	0.0929±0.0212
Bone	0.0616±0.0019
Lung	0.0522±0.0150
Kidney	0.0470±0.0143
Pleura	0.0434±0.0049
Pancreas	0.0360±0.0097
Spleen	0.0286±0.0057
Heart	0.0262±0.0088
Thyroid	0.0233±0.0076
Brain	0.0230±0.0092
Peritoneum	0.0211±0.0122
Pericardium	0.0203±0.0071
Diaphragm	0.0192±0.0031
Large Intestine	0.0141±0.0033
Skin	0.0140±0.0071
Small Intestine	0.0098±0.0019
Gallbladder	0.0097±0.0007
Stomach	0.0081±0.0019
Omentum	0.0068±0.0030
Skeletal Muscle	0.0032±0.0013
Bladder	0.0020±0.0025
Uterus	0.0020±0.0025
Vagina	0.0017±0.0012
Prostate	0.0017±0.0009

27 target sites and their self edge weights (ensemble average) listed in decreasing order.

The remaining 7 sites (below the cut-off, starting from skin) have two-step transition path probabilities that are equal to or more probable than their direct one-step path from lung (taking into account standard deviations). We call these the ‘second-order’ sites. The interpretation of these sites is if there is a metastatic tumor at one of these sites, it is equally probable, or more probable that there is also a metastatic tumor at an intermediate site, most probably the lymph nodes (reg) or adrenal gland. Skin is the most significant second-order site, suggesting a possible pathway from a primary tumor in the lung to a metastatic tumor on the skin via the lymph node (reg) or adrenal gland (not shown, but almost as probable).

**Table 4:** Mean first-passage times from lung.

Target Sites	MFPT (unnormalized)	MFPT (normalized)
Lymph Nodes (reg)	5.6414±0.4919	1.0000±0.0872
Lymph Nodes (dist)	8.3541±0.8096	1.4809±0.1435
Adrenal	10.0349±1.0068	1.7788±0.1785
Liver	10.6139±1.0226	1.8814±0.1813
Lung	13.0284±1.1497	2.3094±0.2038
Bone	16.0277±1.4508	2.8411±0.2572
Kidney	20.3944±1.9664	3.6151±0.3486
Pleura	22.9329±2.4375	4.0651±0.4321
Pancreas	26.4350±2.6438	4.6859±0.4686
Spleen	33.7009±3.4925	5.9739±0.6191
Heart	36.5513±3.6359	6.4791±0.6445
Brain	40.5540±4.3179	7.1886±0.7654
Thyroid	41.3240±4.0700	7.3251±0.7215
Pericardium	46.8599±4.1645	8.3064±0.7382
Diaphragm	51.3372±5.6196	9.1001±0.9961
Peritoneum	51.9555±5.4518	9.2097±0.9664
Large Intestine	69.0501±7.3192	12.2399±1.2963
Skin	79.2006±8.4505	14.0392±1.4979
Gallbladder	104.9654±10.0373	18.6063±1.7792
Small Intestine	105.8723±9.9567	18.7670±1.7649
Stomach	122.4070±12.7034	21.6980±2.2518
Omentum	155.6364±15.8049	27.5883±2.8016
Skeletal Muscle	313.7172±30.6400	55.6098±5.4313
Bladder	620.7585±63.7243	110.0362±11.2958
Prostate	630.6260±68.4618	111.7854±12.1356
Vagina	630.8929±64.6222	111.8327±11.4550
Uterus	633.1578±63.9966	112.2342±11.3441

Mean first-passage times (unnormailzed and normalized) from lung to each target site, obtained by Monte Carlo simulation. Histogram plot is shown in Figure 12.

The classification of sites allows us to quantify possible disease progression paths (described in terms of ‘random-walkers’) from lung to a given metastatic location. This is shown in Figure 9 where we focus on the multiple pathways by which cancer can spread from a primary lung tumor to the liver. We show in the figure the outgoing connection from lung to liver (with weight 0.08028±0.00946), since liver is a first-order site. Roughly 92% of the random walkers, however, do not transition to liver on the first step, but go instead to a different first-order site. Some of these will pass to the liver on the second step, as shown by the directed (solid) arrows. Still others pass to a second-order site, and then to the liver, as shown by the directed (dashed) arrows. In this way, all possible pathways to the liver from lung can be compared probabilistically and one can make quantitative predictions on which other sites might have metastases if a lung cancer patient develops a metastatic liver tumor.

**Figure 10:**
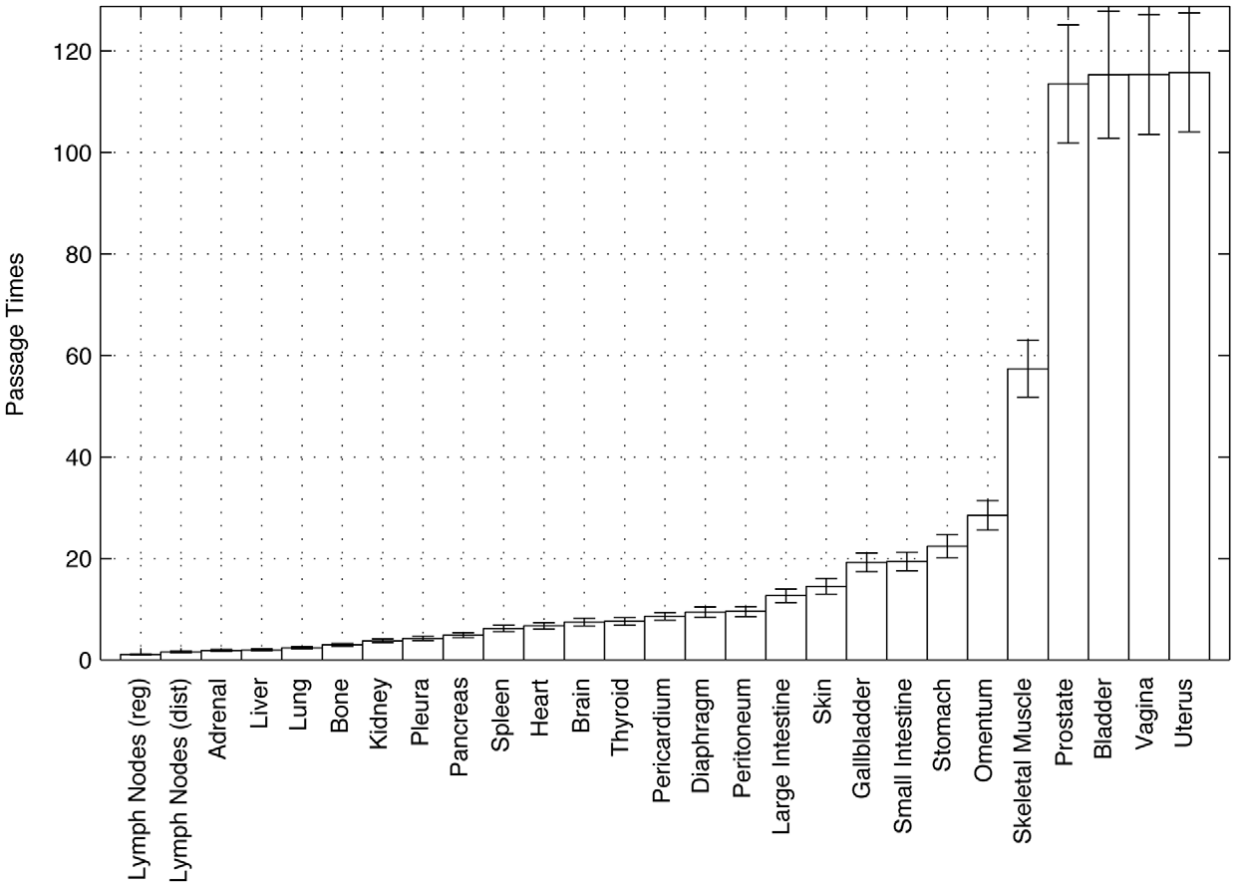
Mean first-passage time histogram for Monte Carlo computed random walks all starting from lung. Error bars show one standard deviation. Values are normalized so that lymph node (reg) has value 1, and all others are in these relative time units.

### Self-seeding Sites

A recent focus in the literature has been on the possibility that tumors can ‘self-seed’ (see [31], [33]) since that process would help explain the exceptionally rapid (‘Gompetzian’ [34]) growth of certain primary tumors. In addition, these papers discuss the possibility, not yet proven experimentally, that self-seeding could potentially occur from a metastatic site back to itself, i.e. ‘metastasis re-seeding’. The focus on self-seeding of the primary tumor (circulating tumor cells that colonize their tumors of origin) demonstrated convincingly in mouse models [33] has led to the general concept that cancer progression, and hence progression pathways, may not be a strictly uni-directional process of progression from primary tumor to sequentially distant metastatic sites. It may well involve aspects that are more multi-directional in nature, such as tumor self-seeding, re-seeding of the primary tumor from a metastatic tumor, or re-seeding of a metastatic site from the metastatic tumor. Experimental evidence and the development of theoretical models that support this, is currently an active area of research. In our model, a site that is self-seeding is one in which a random-walker leaving that site can return directly. The simplest way (but not the only way) to do this would be after one step, if the site has an edge connecting back to itself. This would correspond to a non-zero probability in the diagonal entry of the transition matrix. We list in Table 3 the sites that have this property, along with the edge weighting, listed from strongest to weakest. For primary lung cancer, the most strongly weighted self-connecting edges are the lymph nodes (reg and dist), liver, adrenal, bone, and lung. A more thorough analysis of this potentially important multi-directional mechanism of progression for each given type of primary cancer, along with the average time it takes to self-seed will be the topic of a separate publication.

**Figure 11:**
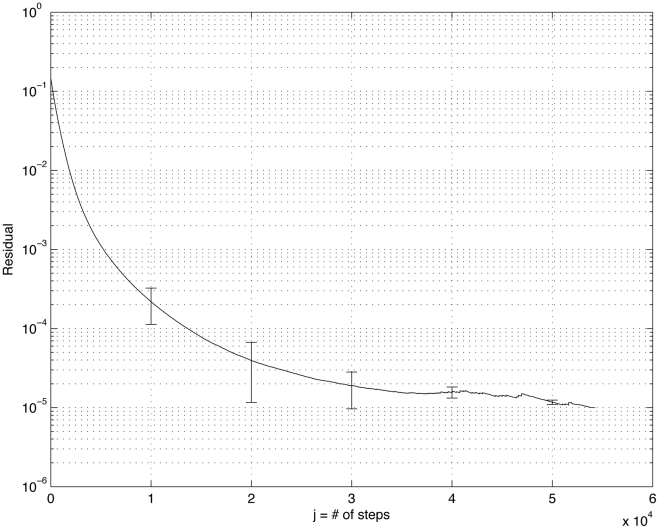
Ensemble convergence to 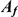, starting from 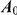. y-axis is 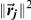
z, x-axis is step *j*. We use an ensemble of 1000 trained matrices 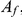 each conditioned on the same initial matrix 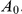 The average convergence curve is shown, along with standard deviations marked along each decade showing the spread associated with the convergence rates.

### Mean First-passage Times

An important quantity associated with our model is called ‘mean first-passage time’ to each of the sites – how many steps, on average, does it take for a random walker to pass from the lung site to each of the other sites. This gives us a model based timescale (not limited to take on discrete values) associated with disease progression, something a static autopsy data set cannot give us directly. It is important to keep in mind that these values are model based only, they do not arise from comparisons of disease time histories, something that could be done with a different data set that contains time progression information. To calculate these times, we follow a random walker starting at the lung position, progressing from site to site until all of the sites have been visited at least one time. Using this method for roughly 10,000 of these random walkers, we collect statistical information on the mean first-passage time to each of the sites, i.e. the average number of steps it takes to first arrive at each site. We show below in Table 4 the mean first-passage times from the lung site, which we obtain by Monte Carlo simulations using an ensemble of 10,000 realizations, where each realization is run long enough in time so that all sites identified by the lung cancer target vector are visited at least once. We emphasize that the mean first-passage times are distributed over a range of positive values quite distinct from the discrete values required in the underlying Markov process.

**Figure 12:**
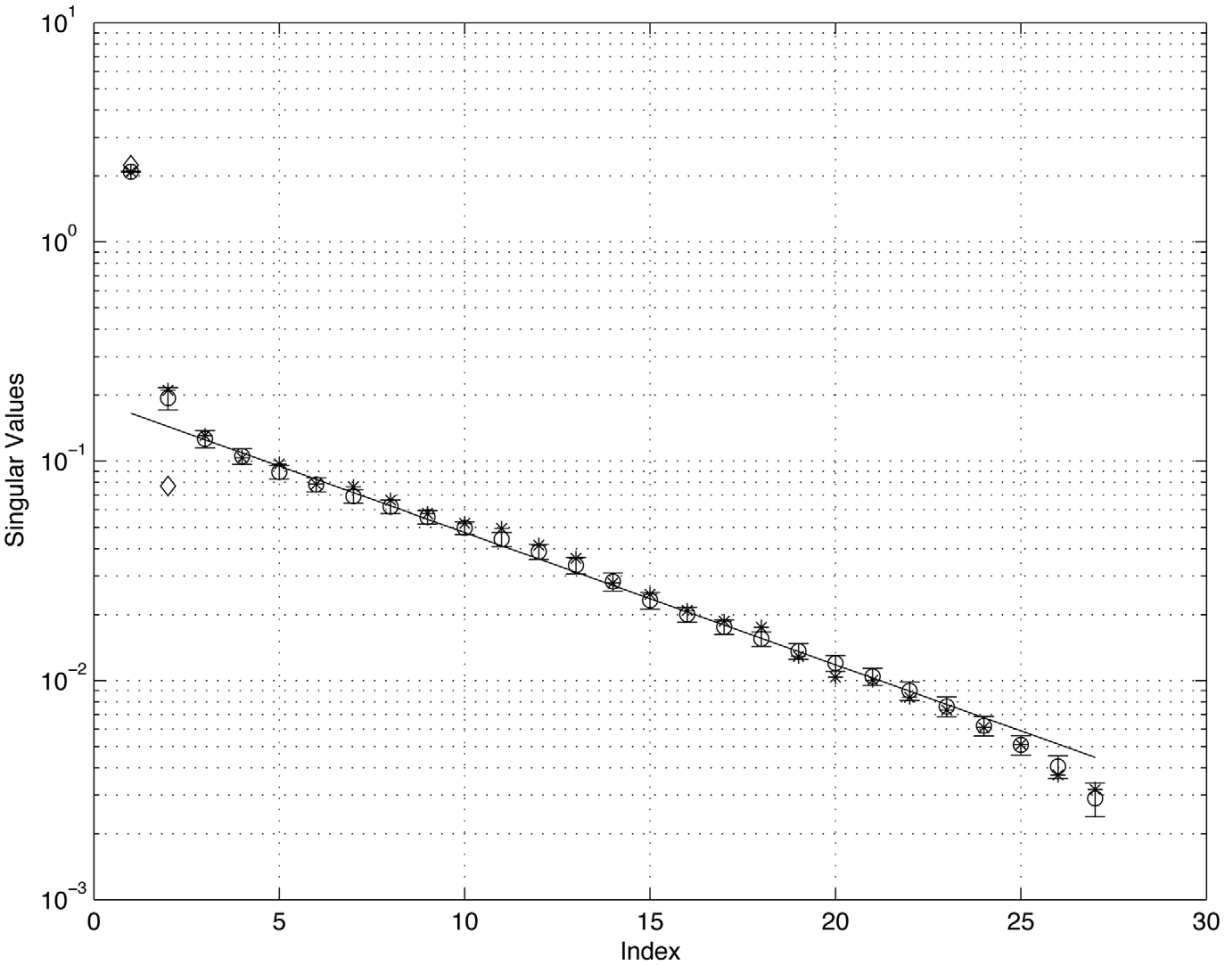
Average distribution of the 27 non-zero singular values associated with the ensemble of 1000 matrices 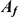 all obtained using the same 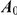. *x*-axis is the index *n*, *y*-axis is 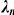. Data points (open circles) indicate the sample average, with error bars showing the sample standard deviations. Line is a least squares curve fit through 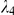 through 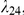 showing linear decrease with exponent 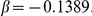 The 27 non-zero singular values reflect the fact that there are 27 entries in the steady-state target distribution for primary lung cancer. The two diamond shaped data points are the two singular values associated with the initial matrix 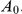 The 27 ‘asterix’ data points are those obtained from a trained matrix using a perturbed 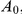 with Rank 2 perturbation. See text for details.

Despite the fact that these mean first-passage times are model-based (i.e. time passage information is not directly in the data set) they are interesting from several points of view. The normalized values, shown in the right column of the table, are obtained by dividing each entry of the un-normalized column by the lymph node (reg) passage time time of 5.6414. This way, everything is measured with respect to the time associated with the progression from lung to regional lymph nodes, providing a relative predictive timescale for average progression. If a patient with a primary lung tumor progresses to a metastatic tumor in the regional lymph nodes after one year, one might expect it to take roughly another 6 months to progress to the distant lymph nodes, or roughly 9 months to the adrenal gland. The interpretation is not that the disease will spread from lung to lymph nodes to liver to adrenal, etc. all in one individual patient (since the model is based on an ensemble data set), but that one, or perhaps several of these secondary sites will eventually produce metastatic tumors, and we have a predictive handle on the progression timescales. The mean first-passage time histogram is plotted in Figure 10 and gives a visual representation of the relative timescales to each of the sites. The sites seem to be grouped into approximately three clusters. In the first group, consisting of sites LN (reg) - Bone, there is an approximate linear increase in the mean first-passage times. The second grouping (Kidney - Peritoneum) also increases linearly, but on a slightly shifted line. The third grouping (Large intestine - Uterus) increases (roughly) exponentially. Sites in this group, with very large mean first-passage times, like prostate or bladder, would be ones in which, if a metastatic tumor does appear, would indicate poor prognosis as other areas would have had a lot of time and ‘probabilistic’ opportunities to develop tumors as well.

Not shown in the table and figure are mean first-passage times from sites other than lung. But it is worth pointing out that we have calculated these times starting at all 50 sites, and the shortest mean first passage time occurs from pleura to adrenal, with a un-normalized time of 1.02, or normalized value of 0.1811. This exceptionally short passage time indicates that if the lung tumor does progress to the pleura, one might expect a short time later for progression to occur to the adrenal gland. As mentioned earlier, this is another possible indication of the potential importance of adrenal gland involvement in lung cancer progression. We are currently comparing our model based mean first-passage times with other data sets that contain the time-history of the disease in individual patients and ensembles.

## Discussion

The computational model we develop and discuss in this paper is an ensemble based Markov chain/random walk model of disease progression in which we use a stochastic transition matrix with entries that are (approximately) normally distributed. The model can help us quantify pathways of progression for lung cancer, and can be used as a baseline model in which to compare more targeted models which use correlations among sites making up the ensemble (i.e. the individual patients making up the ensemble), and use timescale information on disease progression. The model underscores the importance of the complex and heterogeneous nature of the connections among the many potential metastatic locations and bolsters the case for a fairly complex view of the importance of a whole host of subtle connections among sites that may or may not produce clinically detectable tumors, but that seem crucial in the eventual detailed understanding of cancer progression. We believe this autopsy based ensemble study gives important baseline quantitative insight into the structure of lung cancer progression networks that will be useful for future comparisons. Similar techniques can be used for other primary cancer networks. Three key findings based on the model are:

- Metastatic sites can be classified into ‘first-order’ and ‘second-order’ sites based on the comparative values of the one-step vs. two-step transition probabilities. This allows us to lay out the layers of progression from lung to a given site, such as liver, shown in Figure 9 which lays the groundwork for a complete probabilistic classification of all pathways from primary tumor sites to metastatic locations;
- The classification and quantification of ‘self-seeding’ transition values gives us a network based interpretation of some recent biological insights [33] that will be the focus of a separate study on probabilistic mechanisms of multi-directionality;
- Model based mean first-passage times give us relative time information (based on average passage time to regional lymph nodes) about progression that can be used for future comparisons with data sets that contain time progression histories.

An important current direction of this work is to develop ‘data assimilation’ tools that would allow us to incorporate new data (non-autopsy data, individual patient histories, data made up of patients with targeted treatments, etc.) into the ensemble model. The problem is similar to that encountered by the weather prediction community [35] where these techniques have been highly developed and have played a crucial role in going from generic model-based calculations to targeted and accurate short term calculations that focus on prediction and *quantifying the uncertainty* inherent to the predictions [36].

## Methods

Because we are computing the entries of a 50×50 matrix using only the 50 entries of our target steady-state, the solution to this problem is not unique, a problem which is addressed in the works of [37], [38], and [39] for example. In those papers, the solution to this constrained linear inverse problem is obtained by identifying the transition matrix that satisfies a certain maximum entropy condition, and also one obtained by satisfying a least-squares condition. More relevant to our problem is a criterion which targets a family of solutions by pre-conditioning the search on an approximate transition matrix informed by the data, followed by an iteration process which then adjusts the entries until a transition matrix with the correct steady-state is obtained. We show that this process converges, and we use the algorithm to create an ensemble of transition matrices whose entries are best interpreted as (approximately) normally distributed random variables. We then characterize the ensemble of stochastic transition matrices using the means and variances of the singular value distributions [40] associated with the ensemble.

### Algorithm to Compute the Markov Transition Matrix

The three key steps in computing the transition matrix are:

- Step 1 - The choice of initial matrix *A*_0_: First, an approximate transition matrix, 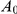, is obtained based on information we extract directly from the data set [6]. For the ‘lung row’ of 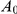, we use the lung target distribution shown in Figure 2(b), which is the metastatic distribution in a population of people with lung cancer primary tumors. This is our first approximation to how the outgoing edges from the lung are weighted. On all of the other 49 rows, we use the generic distribution shown in Figure 2(a). Since we do not know, a priori, how any of the other metastatic sites communicate with any of the others, we use this ‘agnostic’ distribution for all of these non-lung rows. Two key properties of 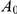 constructed this way are that it has Rank = 2 (i.e. only two linearly independent rows), and it does not have our target distribution shown in Figure 2(b) as a steady-state, hence we know 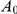 is not the correct transition matrix for lung cancer. Therefore, we perform an iteration process in Step 2 which adjusts the entries of 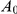 to arrive at a final transition matrix 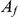 that has higher rank (typically the same rank as the number of entries in the target vector), and has the target distribution (Figure 2(b)) as a steady-state.
- Step 2 - The iteration process to *A_f_*: 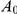, is then used to start an iteration process where the entries are adjusted iteratively, using randomized adjustments, until its steady-state distribution converges to the target distribution. The converged matrix obtained after this process is what we call the ‘trained’ lung cancer matrix, 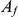. We will discuss this key step further below.
- Step 3 - Creating an ensemble of *A_f_*’s: Because the iterative procedure is based on random adjustments of the matrix entries, and because we adjust the entries only up to some pre-determined numerical value defined as our convergence threshold (typically chosen to be 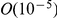), the transition matrices produced from Step 2 should be thought of as having entries that have some inherent probability distribution associated with them, with a sample mean and variance obtained by collecting an ensemble of these matrices. We will show two of the key edge probability distributions (lung to regional lymph nodes, and lung to adrenal) and also discuss the statistical spread of the ensemble of transition matrices using their singular value distributions as a diagnostic tool.

### Convergence of the Algorithm

We now describe Step 2 of our algorithm in more detail, the iterative training stage which takes us from our initial matrix 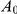, to our final matrix 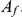 Define the transition matrix after step *j* in the iteration process to be 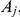 with corresponding steady-state 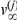 defined as
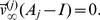
(9)


Our goal is to find the entries of 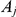 so that
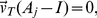
(10)i.e. so that 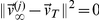. We do this iteratively as follows. Since 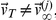 we can define a ‘residual’ at step *j*:
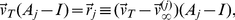
(11)where 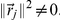 Our goal is to find the entries of 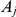 so that 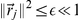 where 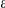 is defined as our numerical convergence threshold. In practice, we do this by calculating 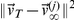 directly and iterate the entries of 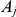 until 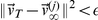, where typically we take 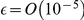.

Stated more generally, our goal is to solve the following linear constrained optimization problem. Given a target vector 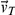, find the entries 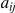 of the matrix *A* to minimize the Euclidean norm of the residual vector 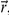 where:
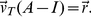
(12)


The constraints are 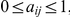 and 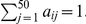 Most importantly, we have pre-conditioned the iterative process in Step 1 on our particular initial matrix 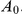 The general framing of this problem as a constrained optimization problem is discussed in [37]–[39].

To do this, we iteratively adjust the entries of 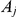 at each step (so as to maintain the constraint that all rows sum to one) according to the following algorithm:

1. Calculate the residual 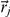 at step *j*, starting with 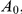 (*j* = 0);
2. Pick the column of 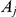 corresponding to the maximum entry of 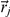;
3. Pick the column of 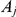 corresponding to the minimum entry of 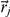;
4. Pick a row of 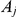 at random;
5. Decrease the entry of 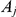 selected in step (ii) by 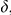 increase the entry of 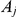 selected in step (iii) by 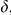 where 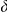 is scaled with the size of 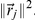 This new matrix is 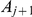;
6. Calculate the new 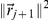 and stop if 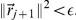 Otherwise go to step (ii) and repeat the process.

Because of the randomized nature of the algorithm, and because of the finite threshold of convergence, the converged final matrix 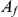 will be slightly different each time the iterative process is carried out, even when all the trained matrices start with the same initial 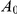. Thus, we carry out the iteration and convergence process, producing an ensemble of 1000 final transition matrices 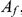 and we show the convergence (down to 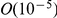) of the ensemble in Figure 11 (plotted on a semi-log plot). The solid curve is the average convergence rate computed from the 1000 training sessions, while the error bars show the standard deviations associated with the ensemble, showing the spread of the convergence rates, which are relatively tight.

### Singular Values and Properties of the Ensemble

A very useful diagnostic tool to characterize the structure and understand the statistical spread associated with the matrices in the ensemble are the singular values, (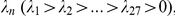 associated with the collection of 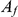
’s. These are shown in Figure 12, plotted from largest to smallest. Values shown (as open circles) are the sample means associated with the singular values of the ensemble of 1000 converged matrices 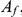 all trained using the same initial matrix 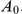 The error bars show the sample standard deviations, which are small. The 27 non-zero singular values reflect the fact that there are 27 entries in the steady-state distribution for primary lung cancer. An equivalent way to say this is that the rank of 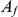 is 27, while the nullspace dimension is (approximately) 23. The standard deviations show the statistical spread associated with two sources of uncertainty, one is the random search algorithm we use to obtain convergence, and the other is the convergence threshold, which we typically take to be 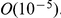 The small standard deviations indicate that the algorithm is converging to the same final 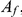 within a relatively small range of statistical spread. On this graph, we also show the least squares curve fit to singular values 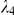 through 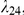 which follow a slope 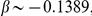 indicating that the singular values roughly decrease like 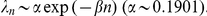 The two diamond shaped data points on the graph correspond to the two singular values of 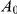 reflecting the linear independence of the two distributions from Figure 2 that we use in 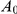. We point out that the 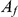
’s should not be viewed as small perturbations of 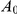 - our convergence algorithm starts with a rank 2 matrix and generates an ensemble of (approximately) rank 27 matrices all within a relatively tight statistical spread.

We also show one other set of singular values on the graph with the asterix data points. To test the robustness of the ensemble with respect to perturbations of the initial matrix 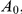 we start the search with an initial matrix of the form 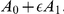 Here, the perturbation matrix 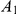 is a 50×50 rank 2 matrix obtained by giving each entry in the lung row a uniformly distributed random number in the interval [–1,1], and each entry in all the other rows another uniformly distributed random number in the interval [–1,1]. This creates a random rank 2 matrix. The perturbation parameter 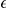 is chosen so that the perturbation size is (roughly) 5% as compared with the average row value of 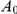. The asterix data points, which correspond to a converged 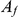 below a threshold of 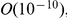 all fall within the one standard deviation bars of the unperturbed values, again showing that the final converged matrix is relatively robust to small changes in the initial matrix 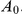 For definiteness, when we make conclusions associated with Monte Carlo simulations, we use the ensemble averaged set of 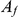
’s obtained over a set of 1000 converged matrices, each converged to within 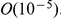 Because of this, we view the transition probabilities of the Markov chain, i.e. the edge values in our network, as themselves being random variables, with a standard deviation that we can characterize.
